# A Common Family Factor Underlying Language Difficulties and Internalizing Problems: Findings From a Population-Based Sibling Study

**DOI:** 10.1177/0022219420911634

**Published:** 2020-03-24

**Authors:** Siri Saugestad Helland, Espen Røysamb, Ragnhild Eek Brandlistuen, Monica Melby-Lervåg, Kristin Gustavson

**Affiliations:** 1 Department of Child Health and Development, Norwegian Institute of Public Health, Oslo, Norway; 2 Regional Centre for Child and Adolescent Mental Health (RBUP), Eastern and Southern Norway, Oslo, Norway; 3 Department of Psychology, PROMENTA Research Center, University of Oslo, Norway; 4 Department of Special Needs Education, University of Oslo, Norway; 5 Department of Mental Disorders, Norwegian Institute of Public Health, Oslo, Norway

**Keywords:** language difficulties, internalizing problems, family factor, comorbidity, MoBa

## Abstract

Studies have identified concurrent, longitudinal, and bidirectional associations between language difficulties and internalizing problems. This is commonly explained by social exclusion or withdrawal from peers, but underlying mechanisms are not well understood. This study uses sibling data to investigate if the comorbidity between language difficulties and internalizing problems is best explained by familial factors shared by siblings, such as genes or family environment, or nonfamilial factors specific to each child, such as peer environment. Data include 5,568 siblings at 5 years and 3,654 siblings at 8 years participating in the Norwegian Mother, Father and Child Cohort Study (MoBa). We constructed a latent factor model at 5 and 8 years, including a family comorbidity factor capturing correlations between language and internalizing problems that were equally strong between as within siblings. Results showed that the correlation between one sibling’s internalizing problems and the other sibling’s language problems was mostly accounted for by a family comorbidity factor. The best-fitting longitudinal model included stability of the family comorbidity factor and stability of language and internalizing problems within each sibling and no cross-sibling or cross-trait longitudinal associations. This suggests that the association between language and internalizing problems may be best explained by family factors.

There is substantial evidence of an association between early language difficulties and later internalizing problems, such as anxiety and depression (Brownlie et al., 2016; Yew & O’Kearney, 2013). The association is found for different types of language difficulties (Conti-Ramsden & Botting, 2008) and at different ages (Beitchman et al., 2014). Clinical studies have shown concurrent comorbidity rates of 50% to 70% (Benner et al., 2002), while longitudinal studies have shown that children with specific language impairment (SLI) are twice as likely as their peers to develop clinical levels of emotional problems (Yew & O’Kearney, 2013). Recent evidence from population-based samples is mixed regarding a reciprocal association between language difficulties and internalizing problems (Bornstein et al., 2013; Helland et al., 2018).

There are several lines of explanations for the association between language difficulties and internalizing problems. Comorbidity may be explained by problems in one area causing problems in another area, directly or indirectly, or by a common underlying factor explaining problem in both areas (Willcutt, 2014). Research on the association between language difficulties and internalizing problems has mainly been based on causal assumptions. It has been suggested that children with language difficulties may be excluded or withdrawn from play (Liiva & Cleave, 2005), which again may lead to emotional problems (Yew & O’Kearney, 2013). Language difficulties may also cause problems in learning emotion regulation from caregivers (Fujiki et al., 2002). This could be due to parents adjusting their level of emotion regulation to a child’s language level (Stansbury & Zimmermann, 1999), or due to problems in the development of inner speech (Lidstone et al., 2012). Others again suggest that emotional activation or emotional problems may compromise a child’s capacity to learn and improve their language (Moilanen et al., 2010).

Family factors have, however, been shown to be central for both the development of internalizing problems (Tandon et al., 2009) and language difficulties (Bishop, 2006), suggesting the possibility that the comorbidity between these phenomena could be explained by a common underlying family factor. In the current study, we include all the variance common between siblings in a latent comorbidity factor. In this way, we may investigate how much of the variance is left after the common family variance is accounted for.

## Internalizing Problems

Prevalence estimates of preschool internalizing problems range from 2% to 15% (Egger & Angold, 2006; Wichstrøm et al., 2012). Children with internalizing problems have a stronger family history of internalizing problems than controls, and internalizing problems are transmitted both through genetic and environmental mechanisms (Tandon et al., 2009). Twin studies have shown heritability estimates of 30% to 80% for depressive symptoms in children (Rice et al., 2002). Risk factors for internalizing problems are negative family environments, parenting style, parental mental health, child temperament, problematic peer relationships, and stressful life events (Whalen et al., 2017).

## Language Difficulties

About 7% to 8% of all children experience language impairment (Norbury et al., 2016). Studies show a strong familial component of language difficulties, with heritability estimates ranging from 50% to 75% for school-aged children (Bishop, 2002). Family characteristics such as parenting style, maternal education, or family socioeconomic status may also affect the development of language difficulties (Bornstein et al., 2013). Levels of language difficulties tend to be stable from 5 years, even for children with additional cognitive, social, emotional, and behavioral problems (Norbury et al., 2017).

## Shared Etiology Explanation

The above evidence suggests that both internalizing problems and language difficulties have a strong family basis. Hence, it is possible that the same family factors explain development of both language difficulties and internalizing problems. Shared etiology has partly been found for language and self-control (Beaver et al., 2008), but we have limited knowledge about the degree to which a shared family factor may explain comorbidity between language problems and internalizing problems.

## The Current Study

In the current study, we use sibling data to estimate a common underlying family factor explaining comorbid language difficulties and internalizing problems. This will provide new knowledge about the degree to which comorbidity between internalizing problems and language difficulties is shared between siblings. We assume that if comorbidity between internalizing and language is due to causality between the two phenomena, correlations between them should be much stronger *within* than *between* siblings. The common underlying factor will be modeled to capture only correlation that is equally strong between as within siblings, whereas residual correlation within each sibling will be allowed in addition to this factor.

The aims of the current study were to

1. Estimate comorbid language difficulties and internalizing problems shared between siblings at 5 and 8 years, by modeling a family comorbidity factor;
2. Examine the stability of this family comorbidity factor from 5 to 8 years, that is, from preschool to school age;
3. Examine the longitudinal association between language difficulties and internalizing problems, over and above what is accounted for by the family comorbidity factor.

## Method

### Participants

Data from the Norwegian Mother, Father and Child Cohort Study (MoBa; www.fhi.no/moba) were used. MoBa is a prospective population-based pregnancy cohort study conducted by the Norwegian Institute of Public Health, including 114,000 children; 95,000 mothers; and 75,200 fathers. Participants were recruited from all over Norway from 1999 to 2008, and 41% of invited women consented to participate (Magnus et al., 2006, 2016). Questionnaire data were gathered at Gestational Week 15 from both parents, and from mothers at Gestational Week 30, and when the child was 6 and 18 months and 3, 5, and 8 years.

The current study is based on version 9 of quality-assured data. The original sample consisted of 41,609 children at 5 years and 32,105 children at 8 years. The lower number of participants at 8 years was partly due to dropout and partly due to the continuous nature of the data collection process. We excluded children based on report from the Medical Birth Registry of Norway (MBRN) with serious malformations, cerebral palsy, hearing problems, or other syndromes all of which are thought to affect a child’s language development (*n* = 5,081 at 5 years), and multiple births (*n* = 1,140 at 5 years). Multiples were not included as zygosity was unknown. In families with more than two participating children, only the two oldest were included. The oldest sibling was treated as sibling one, and the youngest as sibling two. Our final sample consisted of all 5-year-olds in MoBa who had a sibling with available data at 5 years (*n* = 5,568) and all 8-year-olds with a sibling with 8-year-data available (*n* = 3,654). For the longitudinal analyses, only children where both siblings had available data at 5 and 8 years were included (*n* = 1,208). Written informed consent was obtained from all participants upon recruitment. The MoBa has obtained a license from the Norwegian Data Inspectorate and approval from the Regional Committee for Medical Research Ethics.

### Measures

#### Internalizing problems

At 5 years, we used 11 items from the Child Behavior Checklist (CBCL; Achenbach & Rescorla, 2000) to measure internalizing problems, characterized by sadness, withdrawal, or anxious behavior. Seven items were from the anxious and depressed subscale, one from the emotional reactive subscale, and three from the somatic subscale. Mothers rated statements about their child’s functioning as 1 *= not true*, 2 = *somewhat or sometimes true*, or 3 = *very true or often true.* In MoBa, the internalizing items correlate .87 with the full internalizing CBCL scale (Helland et al., 2018).

At 8 years, we used the Short Mood and Feelings Questionnaire (SMFQ) and Screen for Child Anxiety Related Disorders (SCARED). SMFQ is based on the *Diagnostic and Statistical Manual of Mental Disorders* (3rd ed., rev.; *DSM-III-R*; American Psychiatric Association, 1987) criteria of depression (Angold & Costello, 1987), and a 13-item subscale was used. Mothers rated how true items were for their child during the last 2 weeks as 1 = *not true*, 2 = *sometimes true*, or 3 = *true.* SCARED was designed to measure *DSM*-defined anxiety symptoms (Birmaher et al., 1999). A 5-item short scale developed by Birmaher and colleagues (1999 was used in MoBa. Mothers rated statements about their child’s functioning as 1 = *not true*, 2 = *somewhat or sometimes true*, or 3 = *very true or often true.* At 8 years, we combined 13 items from the SMFQ and five items from the SCARED into a mean internalizing variable (range, 1–3).

#### Language difficulties

Language difficulties at 5 and 8 years were measured by a 20-item checklist (Language20Q) developed by Ottem (2009) to identify children with risk of language difficulties. The MoBa data set we used in the study included all items when the children were 5 years old, whereas only the semantic subscale was included when the children were 8 years old. As investigating the longitudinal association between 5 and 8 years was a main aim in the current study, we chose to only include the semantic subscale. We used eight items from the semantic subscale, describing problems with the meaning of words, which may be impaired with regard to both understanding and producing language (items are included in Supplement Material). Mothers rated statements from 1 = *does not fit the child/absolutely wrong* to 5 = *fits well with the child/absolutely right*. The Language 20Q has shown acceptable fit in a confirmatory factor analysis and satisfying ability to identify children with language difficulties in a population-based sample (Helland et al., 2018).

As the scales were constructed to capture children with specific problems, they had highly positively skewed distributions (i.e., most mothers reported no language difficulties or internalizing problems). The variables were, thus, transformed into categorical variables, based on the assumption that ordered categories reflect an imprecise measurement of an underlying normal distribution (Rijsdijk & Sham, 2002). In line with the liability-threshold model, polychoric correlations were used to obtain estimates of the associations between the underlying distributions. We chose five categories to be able to differentiate between children with difficulties and to ensure a difficulty group consisting of approximately 5%, reflecting a group in the population with the poorest functioning. The categories were 1 = *no problems*, 2 = *between 1 and the mean score*, 3 = *between the mean score and one standard deviation from the mean score*, 4 = *from one standard deviation to two standard deviations from the mean score*, and 5 = *above two standard deviations from the mean score*. See Table 1 for the distribution of the cut variables.

**Table 1.** Descriptive Statistics for Continuous and Cut Variables.

	Cronbach’s alpha	Gender	*M*	*SD*	Distribution of cut variables (%)				
Measures					1	2	3	4	5
5 years									
Language difficulties	.85	Boys	1.38***	0.51	37.6	23.8	23.4	8.9	6.2
		Girls	1.28***	0.44	45.9	24.1	20.1	6.4	3.5
Internalizing problems	.66	Boys	1.18***	0.20	29.5	37.2	21.7	7.5	4.1
		Girls	1.19***	0.20	26.8	36.4	24.2	8.3	4.3
8 years									
Language difficulties	.84	Boys	1.35***	0.49	37.2	27.6	21.6	7.1	6.5
		Girls	1.28***	0.43	44.3	27.1	19.2	5.1	4.2
Internalizing problems	.75	Boys	1.15*	0.14	19.6	39.9	26.5	9.0	5.0
		Girls	1.16*	0.16	17.0	40.5	28.7	9.1	4.7

*Note*. Language difficulties measured by semantic subscale Language20Q and internalizing problems measured by CBCL. CBCL = Child Behavior Checklist.

Significant gender differences indicated with *p < .05. **p < .01. ***p < .001.

### Statistical Analyses

**Figure 1.**
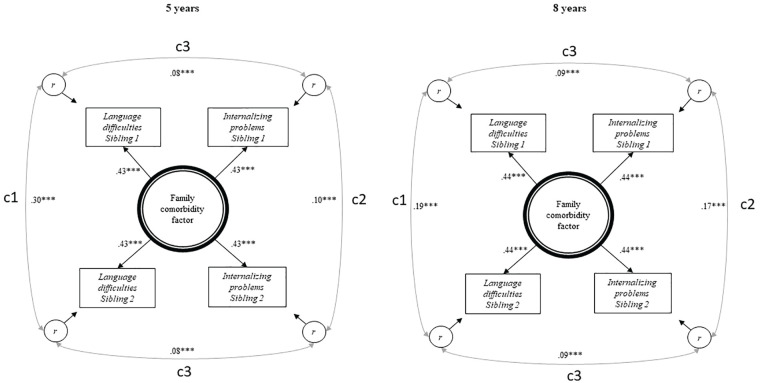
Results from latent factor models showing a family comorbidity factor at 5 and 8 years. *Note.* Latent factor models at 5 years (*n* = 5,568) and at 8 years (*n* = 3,654), reporting standardized correlations and factor loadings. LD = semantic language difficulties measured by Language20Q, IP = internalizing problems measured by CBCL at 5 years and SMFQ/SCARED at 8 years. c1 = correlations representing family similarity specific to language difficulties, c2 = correlation representing family similarity specific for internalizing problems, and c3 = correlation representing comorbidity between language difficulties and internalizing problems unique to each sibling. Family comorbidity factor = correlation that is common for siblings and common for difficulties, r = residual variance, unique for individual child and difficulty, including measurement error. Observed variables are adjusted for gender and child age at return of questionnaire. CBCL = Child Behavior Checklist; SMFQ = Short Mood and Feelings Questionnaire; SCARED = Screen for Child Anxiety Related Disorders. ***Significant at the .001 level.

First, we estimated correlations between language difficulties and internalizing problems across siblings to examine the degree to which comorbidity was shared between children in the same family. Next, a latent factor model was constructed to examine sources of comorbidity that were shared between siblings (i.e., factors making them similar to each other) and sources of comorbidity that were unique to each sibling (see Figure 1). This model has some similarity to A, C, and E models in behavior genetic studies, where A is additive genetic effects, C is shared environment (making children in the same family similar), and E is unique environmental effects (not making children in the same family similar to each other; Røysamb & Tambs, 2016). Factor loadings were constrained to be equal across variables and across siblings, to capture comorbidity within the family. By doing this, we model the degree to which one sibling’s language difficulties predict the other sibling’s internalizing problems to the same extent as their own internalizing problems.

Thus, the family comorbidity factor in the center of Figure 1 represents family factors (A and C in behavior genetic terminology) contributing to comorbidity in both siblings. The left-hand side correlation in Figure 1 (c1) represents family similarity that is specific to language difficulties, while the correlation at the right-hand side (c2) represents family similarity that is specific to internalizing problems. Hence, family resemblance is divided into a family comorbidity factor and two phenotype-specific family factors. As siblings on average only share 50% of their genes, these family factors do not represent all the genetic influences on the traits (unlike behavior genetic models including monozygotic twins).

The top and bottom correlations (c3) represent comorbidity between language difficulties and internalizing problems that is unique to each sibling. These individual specific correlations reflect environmental factors unique to each sibling, but also genetics not shared between siblings (unlike the E in behavior genetic terminology). These correlations, thus, contain all factors that contribute to comorbidity, but that is not shared between two siblings. Potential causality between language difficulties and internalizing problems within each child would be included here. However, our data do not allow distinguishing between causation and shared etiology between the two traits.

**Figure 2.**
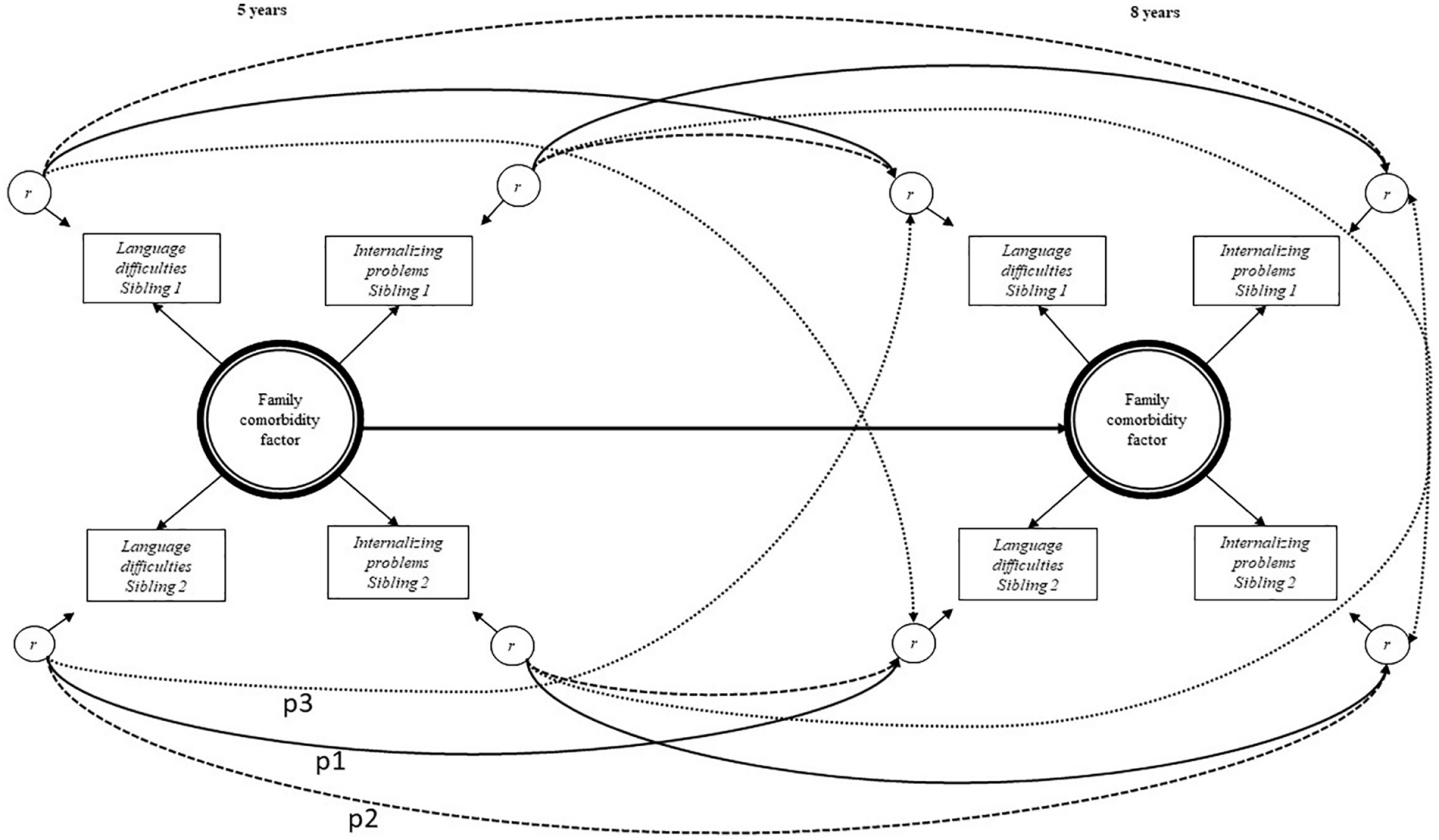
All possible longitudinal pathways tested. *Note.* All longitudinal pathways tested. Paths p1 = within-trait within-sibling longitudinal paths, p2 = cross-trait within-sibling longitudinal paths, p3 = within-trait cross-sibling longitudinal paths. Semantic language difficulties measured by Language20Q and internalizing problems measured by CBCL at 5 years and SMFQ/SCARED at 8 years. Family comorbidity factor = common for siblings and common for difficulties, r = residual variance, unique for individual child and difficulty, including measurement error. CBCL = Child Behavior Checklist; SMFQ = Short Mood and Feelings Questionnaire; SCARED = Screen for Child Anxiety Related Disorders.

Second, the longitudinal stability from 5 to 8 years of the family comorbidity factor was estimated (see Figure 2). Stability of residual variances of internalizing and language problems within each sibling was estimated and notated p1 in Figure 2. Longitudinal paths from each child’s internalizing problems at 5 years to his or her own language problems at 8 years were also allowed (paths notated p2 in Figure 2). In addition, the model included longitudinal paths between each child’s internalizing problems at 5 years to his or her sibling’s internalizing problems at 8 years, and between each child’s language problems at 5 years to his or her sibling’s language problems at 8 years (notated p3 in Figure 2). The fit of this model was compared with the fit of alternative models where the between-trait within-sibling and the within-trait between-sibling longitudinal paths (Paths p2 and p3 in Figure 2) were removed. A good model fit was indicated if the comparative fit index (CFI) and the Tucker–Lewis Index (TLI) was greater than .90 and the root-mean-square error of approximation (RMSEA), .06 or less (Dion, 2008).

The weighted least square mean and variance adjusted (WLSMV) estimator was used for all SEM models due to the use of categorical variables. Probit regression was performed in Mplus, using the WLSMV estimator, which is the default model for ordered categorical data. The model assumes a latent continuous normally distributed variable underlying the observed categorical variable. Standardized output in Mplus gives estimates of the association between a predictor and this latent normally distributed variable and can be interpreted in the same way as standardized estimates from linear models (Gustavson et al., 2019; Muthén & Muthén, 2009). Chi-square values obtained from this estimator cannot be used directly for testing differences of model fit, and the DIFFTEST option was therefore used (Muthén, 2010). This is to test if a more constrained model has significantly poorer fit than a less constrained model. If similar fit, the most parsimonious model is preferred (Bentler & Mooijaart, 1989). As many paths were tested, we applied *p* < .01 as a threshold for statistical significance to reduce the risk of Type I errors. All structural models were adjusted for gender and for the child’s age in months when the mother filled in the questionnaire.

## Results

Table 2 shows that the concurrent correlation between language difficulties and internalizing problems within siblings was modest and significant at both 5 (.23 for sibling 1 and .26 for sibling 2) and 8 years (.27/.28). There were also significant correlations between internalizing problems and language difficulties across siblings at 5 (.18/.19) and 8 years (.19/20). Sibling within and cross-trait correlations at 5 years did not significantly differ between female sibling pairs and male sibling pairs (*p* = .53) nor between the same-sex sibling pairs and opposite-sex sibling pairs (*p* = .07). Sensitivity analyses examining possible rater bias showed that the association between one sibling’s language difficulties and the other sibling’s internalizing difficulties at 5 years (*β* = .179, *p* < .001) did not change substantially when controlling for maternal acquiescence bias (*β* = .175, *p* < .001) or maternal mental health (*β* = .189, *p* < .001/*β* = .180, *p* < .001).

**Table 2.** Polychoric Correlations Between Language Difficulties and Internalizing Problems for Siblings at 5 and 8 Years.

	Sibling 1				Sibling 2			
	5 years		8 years		5 years		8 years	
Measures	LD	Internalizing problems	LD	Internalizing problems	LD	Internalizing problems	LD	Internalizing problems
Sibling 1								
5 years								
Language difficulties	—	.23	.57	.26	.42	.19	.36	.16
Internalizing problems	—	—	.17	.34	.18	.28	.20	.25
8 years								
Language difficulties	—	—	—	.27	.38	.21	.34	.20
Internalizing problems	—	—	—	—	.20	.24	.19	.33
Sibling 2								
5 years								
Language difficulties	—	—	—	—	—	.26	.59	.24
Internalizing problems	—	—	—	—	—	—	.20	.35
8 years								
Language difficulties	—	—	—	—	—	—	—	.28
Internalizing problems	—	—	—	—	—	—	—	—

*Note.* Polychoric correlation between cut variables estimated in Mplus. LD = semantic language difficulties measured by Language20Q and internalizing problems measured by CBCL. CBCL = Child Behavior Checklist.

All correlations significant at the .001 level.

### Family Comorbidity Factor at 5 and 8 Years

Figure 1 shows that the family comorbidity factor accounted for a correlation between language difficulties and internalizing problems of 0.18 (0.43^2^) at 5 years. The sibling-specific correlation between these two traits was .08. Hence, the family comorbidity factor accounts for 69%, 0.18 / (0.18 + 0.08), of the correlation between internalizing and language problems at 5 years. This was very similar at 8 years where the family comorbidity factor accounted for 68% of the correlations between the traits. Hence, the model shows that family factors contribute substantially to comorbidity between language difficulties and internalizing problems. The model had excellent fit at 5 (χ^2^ = 10.26, *df* = 11, RMSEA .000, CFI/TLI 1.000/1.001) and 8 years (χ^2^ = 8.07, *df* = 11, RMSEA .000, CFI/TLI 1.000/1.008). The factor loadings at 5 (.43, *p* < .001) and 8 years (.44, *p* < .001) were substantial. The additional comorbidity, not shared by the siblings (c3), is represented by the correlations at 5 (.08, *p* < .001) and 8 years (.09, *p* < .001). The correlation between siblings’ language difficulties, not explained by the comorbidity factor (c1), was .30 at 5 and .19 at 8 years. The correlation between siblings’ internalizing problems, not explained by the comorbidity factor (c2), was .10 at 5 years and .17 at 8 years. To keep the figure parsimonious, residual variance values for the phenotypes are not shown but are given by the formula 1 − λ^2^ (i.e., 1 minus the squared factor loading on the family comorbidity factor).

### Longitudinal Stability

The current model also allowed direct examination of stability in the family comorbidity factor. By constructing a longitudinal model (see Figure 2), we were able to examine the degree to which family factors contributing to both internalizing and language problems were the same at 5 and 8 years of age. The least constrained model including longitudinal paths within traits within each sibling (Paths p1 in Figure 2), longitudinal paths between traits within siblings (Paths p2), and longitudinal paths within traits between siblings (Paths p3) showed excellent fit (χ^2^ = 47.83, *df* = 48, RMSEA .000, CFI/TLI 1.000/1.000). We then compared the fit of this model with the fit of a more constrained model including only Paths p1 and p2 (χ^2^ = 50.95, *df* = 50, RMSEA .006, CFI/TLI .999/.999). The latter model was preferred, as it was more constrained, and thus more parsimonious, without having poorer fit (Δχ^2^ = 4.65, Δ*df* = 2, *p* > .01). This latter model was then compared with the fit of an even more constrained model, only including Paths p1 (χ^2^ = 56.43, *df* = 52, RMSEA .012, CFI/TLI .996/.994). Again, model fit did not deteriorate in the more constrained model (Δχ^2^ = 6.69, Δ*df* = 2, *p* > .01). Hence, the final model (the best-fitting model) was the model including Paths p1 only.

### The Best-Fitting Model

**Figure 3.**
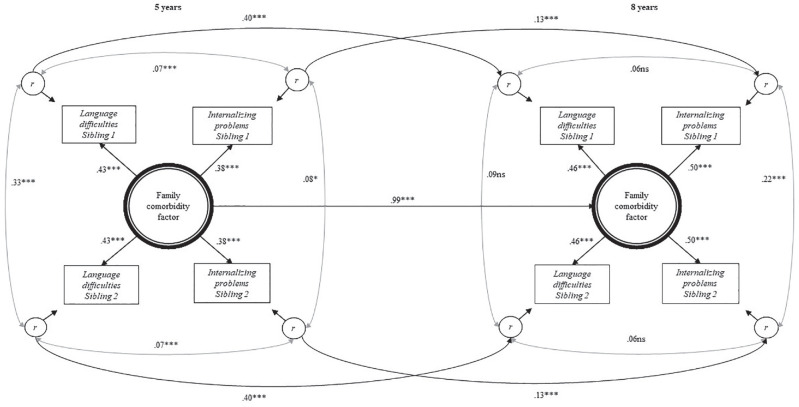
Best-fitting longitudinal model including stability Paths p1 only. *Note.* Standardized results from best-fitting longitudinal model (*n* = 1,208). Semantic language difficulties measured by Language20Q and internalizing problems measured by CBCL at 5 years and SMFQ/SCARED at 8 years. Family difficulty factor = common for siblings and specific for difficulty, r = residual variance, unique for individual child and difficulty, including measurement error. Observed variables are adjusted for gender and child age at return of questionnaire. CBCL = Child Behavior Checklist; SMFQ = Short Mood and Feelings Questionnaire; SCARED = Screen for Child Anxiety Related Disorders; ns = nonsignificant. *p < .01. **p < .05. ***p < .001.

The best-fitting longitudinal model is shown in Figure 3. The shared comorbidity factor had slightly higher loadings at 8 than at 5 years. The stability of this factor over time was, however, .99 (*p* < .001). Hence, there was no evidence of change in what family factors were important for siblings’ shared comorbidity at 5 and 8 years.

This best-fitting model did not contain any longitudinal paths from one trait to another. This suggests that there were no direct effects from one trait to the other over and above what was accounted for by the shared comorbidity factor. There were also no longitudinal paths between siblings, suggesting that siblings’ similarity across time was fully explained by their shared comorbidity.

Stability of language from 5 to 8 years over and above what was accounted for by the shared comorbidity factor within each sibling (p1), was substantial (*β* = .40, *p* > .001), while the corresponding stability for internalizing problems was *β* = .13 (*p* < .001).

## Discussion

The results showed significant correlations between language difficulties and internalizing problems across siblings at 5 and 8 years, suggesting a familial component to the covariance between the areas. This covariance was explored further through a latent family comorbidity factor, with equal loadings from language difficulties and internalizing problems for both siblings. This factor explained a substantial proportion of the comorbidity. When the covariance in language difficulties and internalizing problems shared by siblings was accounted for by the family comorbidity factor, the association between language difficulties and internalizing problems within each sibling was reduced to a minimum.

To our knowledge, no previous studies have directly estimated a common underlying family factor for language difficulties and internalizing problems. In one study, a shared etiology for language and self-regulation was investigated (Beaver et al., 2008), finding evidence for both environmental and genetic shared origins, as well as causal explanations. Family and community predictors of this comorbidity have been identified (Hughes et al., 2016), but the relative importance of family factors compared individual factors was not estimated.

Although our study design did not allow for disentangling genetic versus shared environmental contributions to the sibling correlations, the observed correlations had implications for the maximum heritability involved. As siblings share on average 50% of their genes, the maximum heritability (from additive allele effects) is given by 2r_sib_, or two times the sibling correlation (Plomin et al., 2013). Thus, our findings imply a maximum heritability for language difficulties of .84 (5 years) and .68 (8 years), and correspondingly a heritability for internalizing problems of .56 (5 years) and .66 (8 years). As these figures are in the higher range of previously reported heritabilities, the findings suggest presence of both genetic and shared environmental effects.

In the current study, we found that the stability of the family comorbidity factor from 5 to 8 years was close to 1. The shared comorbidity factor thus comprised the same factors at the two time points. These may be genetic factors with stable effects across 5 to 8 years of age or stable environmental factors in the family. To our knowledge, there are no previous studies investigating the stability of a common family factor for language difficulties and internalizing problems, but previous research has showed both genetic end environmental components for language difficulties and internalizing problems separately (Bishop, 2002; Rice et al., 2002).

As discussed earlier, the shared comorbidity factor only captured covariance between language difficulties and internalizing problems that was equally strong *between* as *within* siblings. Causal effects between language difficulties and internalizing problems are not likely to be equally strong between as within siblings, and the shared comorbidity factor is thus not likely to include covariance between language difficulties and internalizing problems that is due to causality between the two. There were no longitudinal associations between language difficulties and internalizing problems over and above what was accounted for by the common family factor. This indicated that it was more likely that the development of comorbid language difficulties and internalizing problems was caused by common underlying factors, shared by siblings, than direct effects from problems in one area to problems in the other area.

As all observed variables were reported by mother in the current study, we have taken several steps to consider whether rater bias is included in the family factor. Previous studies have showed that mother report is a reliable measure of language difficulties (Helland et al., 2018; Roth et al., 2011). In additional sensitivity analyses, we did not find evidence that maternal response style, in terms of mental health symptoms or acquiescence bias, contributed substantially to the observed associations between language difficulties and internalizing problems in siblings. Nevertheless, we cannot rule out that rater bias to some extent may be included in the family factor.

Previous explanations of this covariance have included that children with language difficulties are at risk of being excluded or withdrawn from play and thus develop emotional problems (Liiva & Cleave, 2005). Our findings do not support this explanation of comorbidity, due to the weak within-sibling between-trait correlation when shared comorbidity was accounted for. Another explanation has been that language difficulties affect the learning of emotion regulation strategies from parents (Fujiki et al., 2002). This explanation could be supported by our results, as parents are shared by siblings. Our results do not, however, contradict results from previous cross-lagged longitudinal studies (Helland et al., 2018; Yew & O’Kearney, 2013), showing that language difficulties *predict* development of internalizing problems over time, and vice versa. The results rather suggest that language difficulties and internalizing problems predict each other longitudinally because they develop in parallel due to shared underlying causes and that interventions may need to target both types of problems directly. Future studies should explore what these underlying mechanisms are, in terms of environmental risk factors, a general ability factor, or in terms of a general psychopathology factor (p-factor; Carver et al., 2017; Caspi et al., 2014).

### Strengths and Limitation

The main strength of the current study is the large population-based sample in a research area traditionally predominated by smaller clinical samples. A second strength is the sibling design enabling us to separate family influences shared by siblings from individual factors.

There are, however, some limitations to the present study. The current design did not enable differentiating between genetic and shared environmental contributions to the family comorbidity factor. Previous studies have shown substantial genetic contribution and modest contributions from shared environment for both language difficulties and internalizing problems. This may suggest that genetic influences are important in the shared comorbidity factor, but this needs to be investigated in further studies with more genetic information than the current. The second limitation is that we could not investigate gender differences. This would lead to too few participants in each group, due to skewed variables and few participants scoring in the lower end of the continuum. As gender differences have been found in previous studies (Helland et al., 2018), this aspect should be a focus in future studies. The third limitation is that we cannot draw conclusions about causality from this observational study. All studies have sources of bias, and findings from different study designs with different sources of bias should be compared to be able to draw stronger conclusions (Keyes et al., 2014). For example, twin studies or randomized controlled intervention studies aimed at preventing language difficulties or internalizing problems versus only one of them may contribute to further understanding of the longitudinal association between language difficulties and internalizing problems.

## Conclusion

The current results accentuate the role of family factors in the development of comorbid language difficulties and internalizing problems. Previous research has proposed withdrawal from peer play and social exclusion as explanations. A reason for this may be that language difficulties and related problems have traditionally been part of pedagogical research, where school and peers are more in focus than family. The current study adds to previous research by showing that this comorbidity may be due to common underlying factors shared in the family, either genetic or environmental, suggesting that future studies need to explore causes of comorbidity shared in the family.

## Supplemental Material

Supplement_material_Language20Q_rev1 – Supplemental material for A Common Family Factor Underlying Language Difficulties and Internalizing Problems: Findings From a Population-Based Sibling Study Click here for additional data file. Supplemental material, Supplement_material_Language20Q_rev1 for A Common Family Factor Underlying Language Difficulties and Internalizing Problems: Findings From a Population-Based Sibling Study by Siri Saugestad Helland, Espen Røysamb, Ragnhild Eek Brandlistuen, Monica Melby-Lervåg and Kristin Gustavson in Journal of Learning Disabilities
