# Supplementary material for: A Common Family Factor Underlying Language Difficulties and Internalizing Problems: Findings From a Population-Based Sibling Study
Source: J Learn Disabil. 2020 Mar 24;53(5):399–409. doi: 10.1177/0022219420911634 (PMC7433392; doi:10.1177/0022219420911634)
Supplement: Supplement_material_Language20Q_rev1 – Supplemental material for A Common Family Factor Underlying Language Difficulties and Internalizing Problems: Findings From a Population-Based Sibling Study [file Supplement_material_Language20Q_rev1.pdf]

Appendix A. Language items used at five and eight years

Semantic language difficulties (Language 20Q)
Forgets words s/he knows the meaning of
Confuses words with similar meanings (e.g. shirt, sweater, jacket)
Has difficulties understanding the meaning of common words
Has difficulties answering questions as quickly as other children
Is often searching for the right words
Uses incomplete sentences
Uses short sentences when s/he answers questions
Has difficulty retelling a story s/he has heard
